# The inhibitory effects of compound Muniziqi granule against B16 cells and harmine induced autophagy and apoptosis by inhibiting Akt/mTOR pathway

**DOI:** 10.1186/s12906-017-2017-4

**Published:** 2017-12-02

**Authors:** Nan Zou, Yue Wei, Fenghua Li, Yang Yang, Xuemei Cheng, Changhong Wang

**Affiliations:** 1 Institute of Chinese Materia Medica, Shanghai University of Traditional Chinese Medicine, The MOE Key Laboratory for Standardization of Chinese Medicines and The SATCM Key Laboratory for New Resources and Quality Evaluation of Chinese Medicine, 1200 Cailun Road, Shanghai, 201203 China; 2 Institute of Experimental Center for Scientific Technology, Shanghai University of Traditional Chinese Medicine, 1200 Cailun Road, Shanghai, China

**Keywords:** Compound Muniziqi granule, Melanoma, Harmine, Autophagy, Apoptosis, Akt/mTOR pathway

## Abstract

**Background:**

Compound Muniziqi granule (MNZQ) is a multi-component herbal preparation and a popular traditional Uighur medicine used in China for treating endocrine disorder-induced acne, chloasma, dysmenorrhea, menopausal syndrome, and melanoma. Harmine presented in MNZQ has been confirmed potential anticancer effect on the B16 cells among others. The purpose of this study is to explore the inhibitory effects of MNZQ against B16 cells and mechanism of autophagy and apoptosis induced by harmine in B16 cells.

**Methods:**

The cell viability was calculated by CCK8 assay. The in vitro tyrosinase activity was determined by spectrophotometry. The harmine-induced autophagy was demonstrated by electron microscopy and MDC staining. Flow cytometry was used to measure cell death and cell cycle distribution. All proteins expression was assessed by western blot.

**Results:**

MNZQ and some herb extracts contained in preparation displayed inhibitory effects on B16 cells but without inhibition on mushroom tyrosinase compared with kojic acid. The formation of autophagosome was markedly induced by harmine with the accretion of LC3-II and the degeneration of p62 in B16 cells, which indicated that harmine was an autophagy inducer. Cell death and sub-G2 population suggested that harmine could induce cell death. Particularly, 3-MA, an autophagy inhibitor, was discovered to prevent harmine-induced decrease of the cell viability and cell cycle arrest on G2 phase, indicating that autophagy was vital to the cell death. In addition, the results indicated that harmine could inhibit the phosphorylation of Akt and mTOR, which might mediate autophagy.

**Conclusion:**

Harmine could induce autophagy and apoptosis by inhibiting Akt/mTOR pathway in B16 cells. Harmine might be a promising therapeutic agent for treatment of melanoma in MNZQ.

## Background

Herbal medicines have gained growing popularity and have been used elsewhere worldwide as alternative and complementary medicine and food supplements [1]. According to the statistics, almost 80% of people use herbs to treat related diseases in the whole world based on their health care needs [1]. In China, mostly 1000 herbs are proven effective and prescribed by TCM practitioners or produced as herbal preparations by pharmaceutical manufacturers [2].

Compound Muniziqi granule (MNZQ), recorded in Pharmaceutical Standards-Uighur Medicine and the Ministry of Health of the People’s Republic of China, is a multi-component herbal preparation and a popular traditional Uighur medicine (TUM) used in China [3]. MNZQ consists of 13 species of plants for medicinal uses, including seeds of *Peganum harmala*, *Cichorium intybus*, *Dracocephalum moldavica*, *Ocimum basilicum*, *Althaea rosea*, and *Nigella glandulifera*; fruits of *Pimpinella anisum*; roots of *Apium graveolens*, *Glycyrrhiza uralensis* and *Cichorium intybus*; cortex of *Foeniculum vulgare*; and herbs of *Matricaria chamomilla* and *Cymbopogon caesius*. Previously study has showed that hormone synthesis and metabolism can be effectively regulated by treating with MNZQ. What is more, it can also treat endocrine disorder-induced acne, chloasma, dysmenorrhea, menopausal syndrome, and melanoma [4].

Melanoma has usually evolved from skin diseases such as chloasma [5]. Despite melanoma has been viewed as a complex disease, the inhibition of tyrosinase is the most common pathway to achieve skin whiteness as it is the key enzyme that catalyzes the rate-limiting step of melanin biosynthesis [6]. Therefore, it has become a focus to search for new drugs from folk medicine which could inhibit tyrosinase activity and decrease the proliferation of melanoma cell.

Apoptosis, or type I programmed cell death, is classified as cell membrane blebbing, cell shrinkage, nuclear condensation and fragmentation, and formation of apoptotic bodies [7]. The mechanism of apoptosis includes the extrinsic and intrinsic apoptotic pathway which results from the activation of caspases (cysteine proteases), which is activated by either death receptor ligation or emission of apoptotic mediators from the mitochondria [8–10]. The tumor cell death is involved in killing cell and inhibiting cell division. During the cell cycle, successful proportion of cells in the G2/M is a critical factor to cell proliferation [11]. Cell cycle regulation imbalance is described in the cancer cells, which promotes the occurrence and development of tumor [12].

Autophagy is a process of self-eating that degrades and recycles cytoplasmic supplies to sustain metabolism and survival of the cell [13]. The formation of notable double-membraned vesicles, named autophagosomes, is one of the characterizing features of this procedure employed to transfer cargo proposed for degradation. Some principal methods are presently used to monitor the induction of autophagy, including electron microscopy, monodansylcadaverine (MDC) staining, biochemical detection of protein LC3-II and P62 [14]. To make a distinction between induction of autophagy and suppression of down-stream steps of autophagy, it was necessary to monitor autophagic flux of autophagy inhibitors. Cell growth, propagation, and angiogenesis are highly associated with the Akt/mTOR pathway [15, 16].

Harmine is a naturally occurring β-carboline alkaloid present in a number of medicinal plants such as *P. harmala* L., *Passiflora incarnata* L. and *Banisteriopsis caapi* (Spruce ex Griseb.) Morton [17]. It has been found that harmine is the most important compound which has been demonstrated to exert strong anticancer activities by suppressing proliferation [18, 19], migration [20], invasion [21] and preventing from tumorigenesis. Harmine can down-regulation the expression of pro-metastatic genes (e.g. MMP-9, ERK and VEGFs) which is related to the foregoing activity, and it was crucial to melanoma cell invasion [22]. Some studies have been reported that harmol (a metabolite of harmine) and β-carboline derivatives could induce autophagy instead of apoptosis [23]. However, harmine has been reported to modulate autophagy and perturb molecular targets of apoptosis, the exact mechanism of harmine-induced autophagy remains unclear.

In the present study, the exciting inhibitory effects of MNZQ and extract from *P. harmala* against B16 cells have been observed. However, MNZQ and extract from *P. harmala* did not exhibit inhibitory effects on tyrosinase activity. MNZQ and the main β-carboline alkaloids harmine among others contained in extract from *P. harmala* showed potential effects on melanoma. The induction of autophagy by harmine in B16 cells was demonstrated by electron microscopy and MDC staining, the expression of LC3-II and p62. In addition, the nuclear morphology was analyzed by hoechst 33,258 assay. Apoptosis rate and cell cycle distribution were detected by annexinV-FITC/PI staining assay and cell cycle analysis. It was identified that 3-MA was found to prevent harmine-induced cell death and cell cycle arrest on G2 phase. Autophagy induced by harmine is mediated by increased autophagy activity and inhibition of the Akt/mTOR signaling pathway.

## Methods

### Chemicals and drugs

Harmine, harmaline, harmane, and harmol (purity > 98%), methylsulfoxide (DMSO), 3-Methyladenine (3-MA), monodansylcadaverine (MDC), L-dopa, hoechst 33,258 and mushroom tyrosinase were purchased from Sigma-Aldrich. Liquiritin, isoliquiritin and glycyrrhizic acid were purchased from Natural Biological Technology Co., LTD (Shanghai). Cell Counting Kit-8 (CCK8, YEASEN, China), bafilomycin A1 (Calbiochem, US), annexin V- fluorescein isothiocyanate (FITC), and apoptosis detection Kit (BD Bioscience, USA) were used. RPMI Medium Modified, fetal bovine serum (FBS), phosphate buffered saline (PBS) and penicillin-streptomycin were obtained from Gibco (Carlsbad, CA, USA). Primary antibodies of GAPDH, LC3, P62, mTOR, p-mTOR, Akt, p-Akt, ERK1/2, p-ERK1/2 were purchased from Cell Signaling Technology (Danvers, MA). MNZQ was offered by Xinjing Uighur Pharmaceutical Co., Ltd. (Xinjiang, China; Batch No.151144). The information, including plant name, herbal name, Chinese name, medicinal parts, formula dosage, and voucher number of 13 species of medicinal plants comprising MNZQ could be referred to our previous study [4].

### Preparation of herbs extracts, MNZQ, and chemicals

The extracts of 13 herbs were prepared according to the preparation process of MNZQ [3]. The 13 dried raw materials (60 g) in MNZQ were pulverized as powder and decocted with 600 mL of water thrice in reflux, each for 2 h, 1.5 h, and 1 h, respectively. The decoctions were combined, filtrated, and concentrated under reduced pressure at 60 °C to afford concentrated extracts (ca. 60 mL). Due to the different extract yield, the 13 concentrated extracts were converted to equivalent amount of raw material concentrations in formula of MNZQ as follow: *P. harmala* 0.8 g/mL, *F. vulgare* 0.8 g/mL, *P. anisum* 2 g/mL, *N. glandulifera* 0.8 g/mL, *M. chamomilla* 0.8 g/mL, *C. intybus* (seed) 0.8 g/mL, *C. intybus* (root) 2 g/mL, *A. graveolens* 0.8 g/mL, *D. moldavica* 0.2 g/mL, *G. uralensis* 0.8 g/mL, *C. caesius* 0.8 g/mL, *O. basilicum* 1 g/mL, *A. rosea* 2 g/mL, respectively. For cell viability test, the 13 concentrated extracts (5 μL) were diluted to 1 mL with culture medium to give concentrations of *F. vulgare*: 4 mg/mL, *P. anisum*: 10 mg/mL, *M. chamomilla*: 4 mg/mL, *C. intybus* (seed): 10 mg/mL, *C. intybus* (Root): 10 mg/mL, *A. graveolens*: 4 mg/mL, *D. moldavica*: 1 mg/mL, *G. uralensis*: 4 mg/mL, *C. caesius*: 4 mg/mL, *O. basilicum*: 5 mg/mL, *A. rosea*: 10 mg/mL. And two herbs were diluted to *P. harmala*: 1 mg/mL and *N. glandulifera*: 2 mg/mL. MNZQ (60 mg) was dissolved in 1 mL water at a concentration of 60 mg/mL. Harmine, harmaline, harmane, and harmol were dissolved in DMSO at a concentration of 40 mM. All of stock solutions were stored at −20 °C until use.

### Cell culture

B16-F-10 melanoma cells (B16 cells) were gained from the Cell Bank of the Chinese Academy of Sciences (Shanghai, China). B16 cells were cultured in RPMI 1640 medium supplemented with 10% fetal bovine serum (FBS) with 100 U/mL penicillin/streptomycin in a dynamic incubation system at 37 °C under an atmosphere of 5% CO_2_ (Thermo Fisher Scientific, USA). When the cells were in the logarithmic growth phage, the cells were used for the following experiments.

### Chemical analysis of MNZQ

**Fig. 1:**
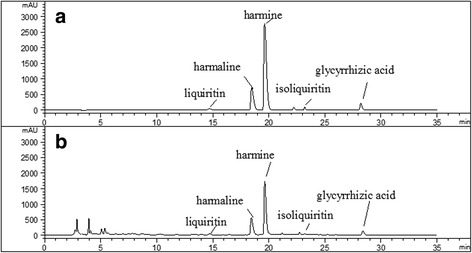
The typical HPLC fingerprint chromatograms of reference standards of liquiritin, harmaline, harmine, isoliquiritin and glycyrrhizic acid (**a**) and MNZQ sample (**b**)

A high performance liquid chromatography (HPLC) fingerprinting of MNZQ has been established previously [4] and eight characteristic peaks were identified as chlorogenic acid, caffeic acid, ferulic acid, liquiritin, harmaline, harmine, apigenin 7-O-glucoside, and isoliquiritin in MNZQ dissolved in 0.05 M hydrochloric acid solution and extracted with ethyl acetate. In order to reducing interference of impurity and accurately determine the contents of targeted markers, including liquiritin, harmaline, harmine, isoliquiritin and glycyrrhizic acid, the separation condition of HPLC and sample preparation of MNZQ have been modified reasonably (unpublished data). The 12 g aliquot of MNZQ sample was directly dissolved in 20 mL water and extracted three times with 30 mL of n-butyl alcohol saturated by water in a 125 mL separatory funnel. The organic supernatants were combined to evaporate to dryness and then the residue was dissolved in 5 mL of methanol. The solution was filtered through a Millipore filter (0.45 μm) to obtain the sample solution before injection into LC system for analysis. The sample was separated on a C_18_ chromatographic column (4.6 mm × 250 mm, 5 μm, Boston Lunna Clone, Boston Analytics, Inc., USA) maintained at 30 °C. The mobile phase was consisted of acetonitrile (A) and ammonium acetate buffer (B) at a flow rate of 1 mL/min, and eluted with gradient elution: 0–10 min (19% A), 10–20 min (33% A), 20–35 min (33% A). The injection volume was 10 μL and the detection wavelength was set at 254 nm. The typical chromatographic fingerprints of MNZQ and mixture reference standards were deposited in Fig. 1. The contents of targeted markers liquiritin, harmaline, harmine, isoliquiritin and glycyrrhizic acid were determined as 0.11, 0.43, 0.21, 0.03, and 0.14 mg/g respectively.

### Anti-melanoma activity of MNZQ and single herb extracts

#### Cell viability

The effects of MNZQ and herb extracts on cell growth were evaluated by the CCK8 kit. The B16 cells were seeded in a 96-well plate with the density of 5 × 10^3^ cells/well in the incubator. The cells were treated with above-mentioned concentrations of herb extracts, MNZQ (60 mg/mL), harmine, harmaline, harmane, and harmol (50, 100, 150, 200 μM) for 24 h. After the addition of 10 μL CCK8 solution/well, they were incubated for 2 h. A microplate reader (BioTek, USA) was used to measure the absorbance at a wavelength of 450 nm.

### Tyrosinase assay

Spectrophotometry was used to detect tyrosinase activity with minor modification [24]. The incubation mixture (150 μL) consisted of 50 U/mL mushroom tyrosinase in PBS (50 μL), 5 mM L-dopa solution (50 μL), stock solutions of individual herb extracts (50 μL) or PBS (50 μL). The mixture was incubated at 37 °C for 10 min and then the absorbance was measured at 475 nm with a microplate reader (BioTek Instruments, Inc., USA).

### The autophagy and apoptosis of harmine-induced

#### Cell morphology

The B16 cells were seeded in culture plates and allowed to attach overnight. The morphology of B16 cells, with or without 40 μM harmine treatment, was evaluated by TEM. Briefly, the cells were immediately fixed in 3% osmium tetroxide in 4 °C for 2 h, along a rinse with distilled water. Dehydrate them in different graded ethanol series then cultured in araldite (Fluka, Buchs, Switzerland). The micrometer thick blocks were stained with toluidine blue, taken photos by a microscope for further observation. They were then trimmed at interest areas to recognize the morphological changes compatible with autophagy. Cut ultrathin into 60–90 nm thick by a diamond knife for the research of electron microscopic. The steps above were mounted on copper grids, double stained in uranyl acetate as well as lead citrate, checked by electron microscope (FEI Company, USA). Take micrographs for qualitative description. All samples underwent the same fixation and processes.

### Autophagosome formation assay

The B16 cells were seeded in 96 well-plates with the density of 5 × 10^3^ cells/well and culture plates in the incubator. The cells were cultured with different concentrations of harmine (40, 50, 60, 80 μM) for 24 h, in order to quantifying the autophagy induction. Cells were cultured in 50 μM MDC for 15 min at 37 °C at last, then washed with PBS (pH 7.4) and the fluorescence measured and photographed by a multimode plate reader (Ex 340 nm and Em 535 nm, Thermo Scientific, USA, OLYMPUS, Japan). All data were performed with three independent, replicate experiments.

### Cell viability

In order determining whether the cell death is connected with autophagy, four B16 cells groups were divided, including control group (medium, only), harmine group, 3-MA group, harmine combined with 3-MA group. Among them, the harmine combined with 3-MA group was treated with 3-MA (5 mM) for 1 h previously. The cell viability was evaluated as above.

### Hoechst 33,258 staining

The B16 cells were seeded in culture plates and allowed to attach overnight. After being subjected to harmine (80 μM) treatment for 24 h, cells were fixed with 4% paraformaldehyde at room temperature for 30 min, then washed with PBS and stained with hoechst 33,258 (50 μg/mL) at 37 °C for 20 min in dark. After incubation and washed by PBS, the nuclear morphological changes of the cells were assessed by fluorescence microscopy (OLYMPUS, Japan).

### AnnexinV-FITC/PI staining assay

The B16 cells were seeded in 6-well plates and allowed to attach overnight. The cells were cultured with various concentrations of harmine (40, 60, 80 μM). After 48 h incubation, cells were harvested and washed with PBS in cold twice. All cells were re-suspended in 300 μL binding buffer, then added by 5 μL of annexin V-FITC (fluorescein isothiocyanate) (2 mg/mL). Incubated for 15 min in dark, 5 μL of PI (propidium iodide, 20 μg/mL) was added for 5 min. BD FACS Calibur flow cytometer (Becton & Dickinson Company, Franklin Lakes, NJ, USA) was performed to analyze the apoptosis of cells.

### Cell cycle analysis

Flow cytometry assay was performed to clarify the distribution of cell period and apoptotic rate. The cells were cultured after treating with harmine (40, 60, 80 μM) for 24 h as described above. After incubation, cells were harvested, washed by phosphate-buffered solution (PBS), and fixed in 70% ice-cold ethanol a night. Then, the cells were incubated with RNase A (100 μg/mL) and PI (40 μg/mL) for 30 min. Use the flow cytometry to determine the DNA content.

In order to investigating whether the cell cycle distribution is connected with autophagy, four B16 cells treatment groups including control group (medium, only), harmine group, 3-MA group, harmine combined with 3-MA group were conducted, in which the harmine combined with 3-MA group was treated with 3-MA (5 mM) for 1 h previously. The evaluation method was as above.

### Western blot analysis

Total protein was extracted by incubation of cell pellet with lysis buffer. The protein concentration was determined by using BCA kit (YEASEN, China) according to the instructions of manufacturer. The cell lysate containing 20 μg of protein was fractionated by SDS-PAGE, transferred to a nitrocellulose filter membrane (Millipore, Billerica, MA, USA). Block them with 5% dried skimmed milk, and incubate the membranes for one night at 4 °C with the appropriate primary antibody. Then, it was incubated with HRP-conjugated secondary antibodies at room temperature for an hour. Protein bands were observed by an ECL chemiluminescence reagent and X-ray film (Tanon 5200, China).

### Statistical analysis

The results were presented according to the means ± standard deviation. Use the one-way analysis of variance (ANOVA) to conduct statistical comparisons. *P* value less than 0.05 were used to show a statistically difference. All data were conducted with three independent and replicate tests.

## Results

### Anti-melanoma activity of MNZQ and herb extracts

**Fig. 2:**
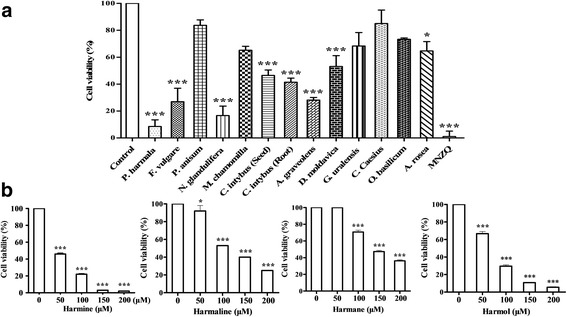
The anti-melanoma effects of MNZQ, herb extracts and active ingredients on B16 cells assessed by CCK8. **a:** *P. harmala*: 1 mg/mL; *F. vulgare*: 4 mg/mL; *P. anisum*: 10 mg/mL; *N. glandulifera*: 2 mg/mL; *M. chamomilla*: 4 mg/mL; *C. intybus* (seed): 10 mg/mL; *C. intybus* (Root): 10 mg/mL; *A. graveolens*: 4 mg/mL; *D. moldavica*: 1 mg/mL; *G. uralensis*: 4 mg/mL; *C. caesius*: 4 mg/mL; *O. basilicum*: 5 mg/mL; *A. rosea*: 10 mg/mL; MNZQ: 60 mg/mL; **b:** active ingredients of harmine, harmaline, harmane, and harmol with concentration ranging from 50 to 200 μM. Results are expressed as the mean ± SD (*n* = 3). ^*^ *P* < 0.05, ^***^ *P* < 0.001 vs. the control group

The anti-melanoma activities of MNZQ and herb extracts were showed in Fig. 2. It could be seen from Fig. 2a that MNZQ (60 mg/mL), the extracts from *P. harmala* (1 mg/mL), *F. vulgare* (4 mg/mL), *N. glandulifera* (2 mg/mL), *C. intybus* (Seed; 10 mg/mL), *C. intybus* (Root; 10 mg/mL), *A. graveolens* (4 mg/mL), *D. moldavica* (1 mg/mL) could inhibit the proliferation of B16 cells. It was found that *P. harmala* and *N. glandulifera* have remarkable anti-melanoma effect with IC_50_ values of 0.90 and 1.04 mg/mL at 24 h,respectively. The ingredients of harmine, harmaline, harmane and harmol contained in *P. harmala* inhibited the proliferation of B16 cells in a dose-dependent manner (Fig. 2b). Among these ingredients, harmine was the most notable effective on anti-melanoma activity with the lowest IC_50_ value of 44.92 μM at 24 h. The IC_50_ values of harmol, harmaline, and harmane were 68.50, 107.8, and 149.7 μM, respectively.

The results of tyrosinase inhibition assay indicated that all of 13 herb extracts and MNZQ did not show any inhibitory effects on tyrosinase at treated concentrations compared with kojic acid (data were not showed).

### The autophagy and apoptosis of harmine-induced

#### The autophagy of harmine-induced

**Fig. 3:**
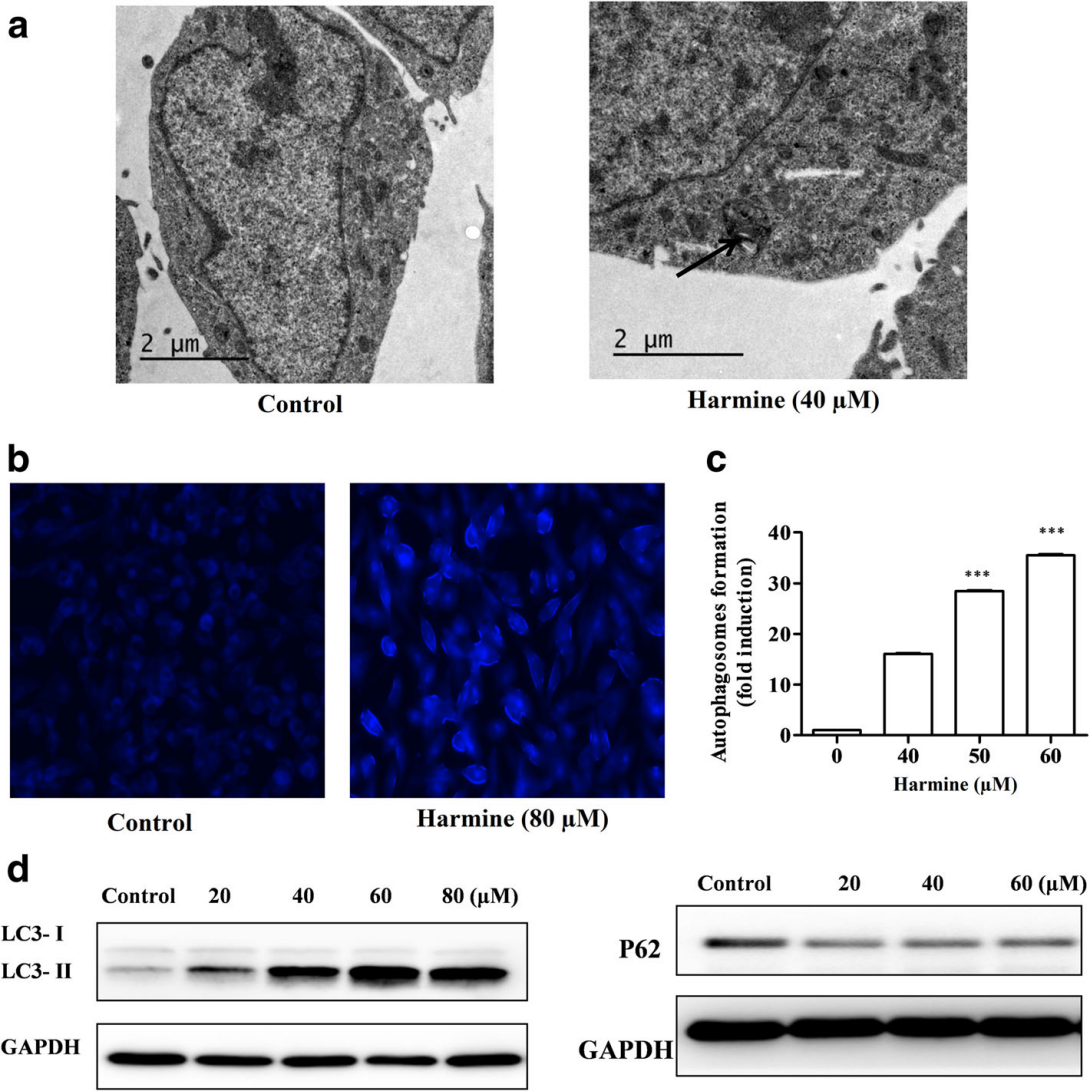
Harmine induced autophagy in B16 cells. **a:** Electron micrographs of normal B16 cells (Control: magnification × 8200) and harmine-treated (40 μM) B16 cells (magnification × 9900); **b:** Fluorescence microscope (magnification × 200) observation on accumulation of MDC-stained autophagic vacuoles of control and harmine-treated (80 μM) B16 cells; **c:** The quantitative analysis of MDC-stained cells of harmine treated (40, 50 60 μM) expressed as the mean ± SD (*n* = 3). ^***^ *P* < 0.001 vs. the control group; and **d:** the autophagy induction evaluated by western blot analysis for LC3-II treated with harmine (20, 40, 60, 80 μM) for 24 h and P62 proteins treated with harmine (20, 40, 60 μM) for 2 h

Electron microscopy was performed to obtain ultrastructural information regarding the autophagic vacuoles in B16 cells. As showed in Fig. 3a, the electron micrograph of harmine-treated in B16 cell showed cytoplasmic phagolysosomes whereas nucleus was normal after treated with harmine (40 μM). In contrast, few phagolysosomes were observed in the non-treated cells.

As known to all, MDC has been considered as a marker for autophagic vacuoles [25]. As depicted in Fig. 3b and c, the harmine treatment led to the accumulation of MDC-stained autophagic vacuoles. The quantitative analysis of MDC-stained cells confirmed that harmine caused a dose-dependent increase. The qualitative analysis confirmed that the control cells showed slight fluorescence, while the cells treated with harmine at 80 μM accumulated MDC into granular structures of high fluorescence intensity.

To measure the occurrence of harmine-induced autophagy, the accumulation of LC3-II and degradation of P62 [26] were assessed by using western blot. The results were followed in Fig. 3d. After treatment of B16 cells with different concentrations of harmine for 24 h, a marked and dose-dependent up-regulation of LC3-II expression was observed compared with the untreated cells. SQSTM1/P62 serves as a link between LC3 and ubiquitinated substrates. After treatment of cells with various concentrations of harmine for 2 h, the levels of p62 presented a declining trend in dose-dependent mode.

### The apoptosis of harmine-induced

**Fig. 4:**
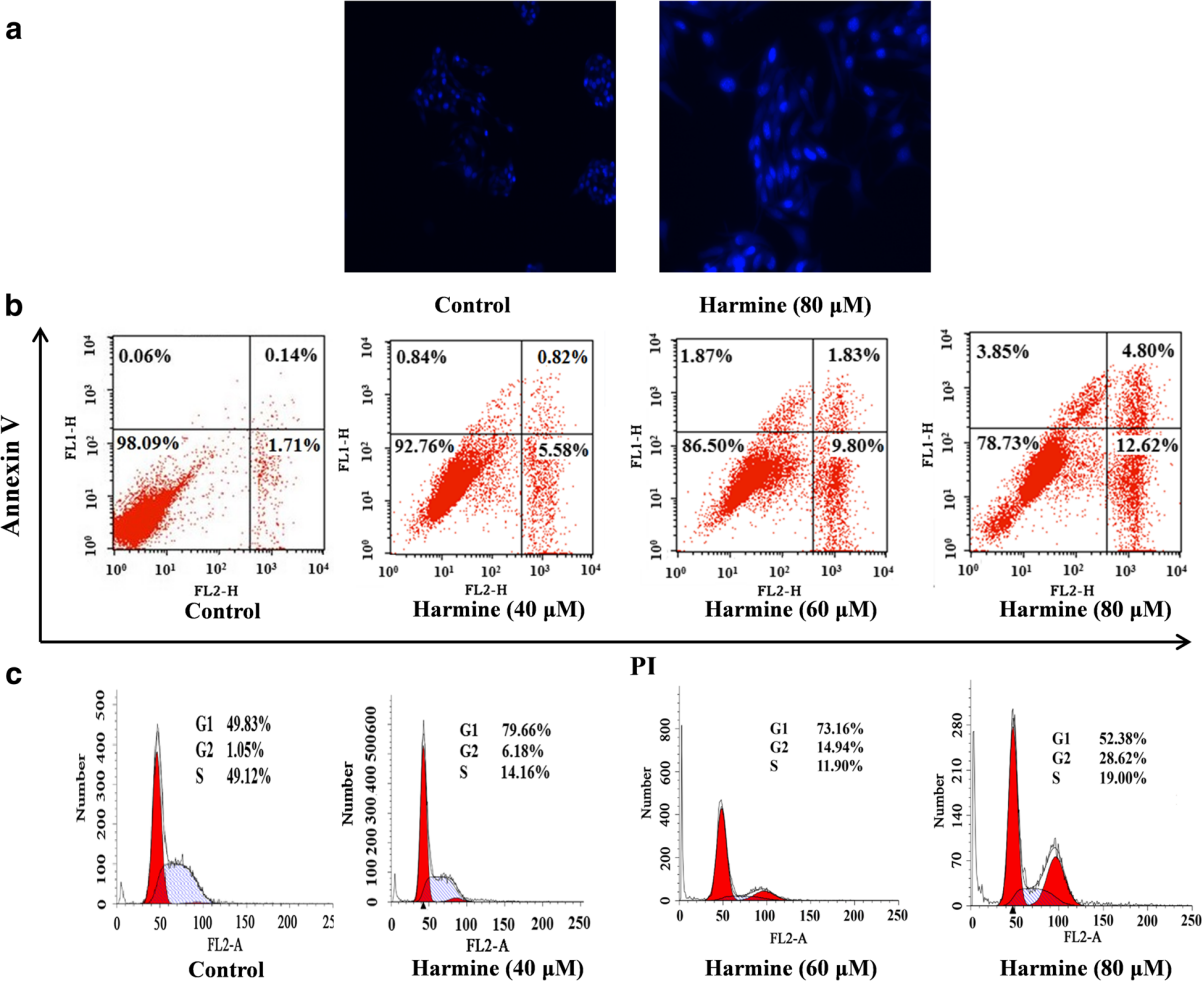
Harmine induced apoptosis in B16 cells. **a:** Fluorescence microscope (magnification, × 200) assessed by hoechst 33,258 staining; **b:** Apoptotic cells detected by flow cytometry with annexinV-FITC and PI double staining; and **c:** Images of harmine induced cell cycle arrest analysis detected by PI staining

Apoptotic nuclear morphology was observed after hoechst 33,258 staining. After treatment with 80 μM harmine for 24 h, as shown in Fig. 4a, the morphology of B16 cells changed, such as the volume of nuclear increased. This change was not the characteristics of the nuclear apoptosis. In the control group, the cells were normal in the morphology.

Apoptosis was also detected by annexin V-FITC/PI double staining assay to distinguish and determine the percentage of apoptotic cells. The proportion of apoptotic cells were followed in Fig. 4b. After harmine treatment, the early apoptotic cells increased from 0.84% (40 μM) to 1.87% (60 μM) and 3.85% (80 μM) and the late apoptotic cells increased from 0.82% (40 μM) to 1.83% (60 μM) and 4.80% (80 μM) when incubated with the indicated concentrations of harmine for 48 h.

Flow cytometry with a PI staining assay was assessed to analyze the effects of harmine on the cell cycle distribution. As depicted in Fig. 4c, the results showed that the accumulation of cells in the G2 phase was from 6.18% (40 μM) to 14.94% (60 μM) and 28.62% (80 μM), compared to the control group (1.05%). These results indicated that cell cycle distribution was significantly arrested in the G2 phase by harmine treatment.

### The relationship between autophagy and apoptosis of harmine-induced

**Fig. 5:**
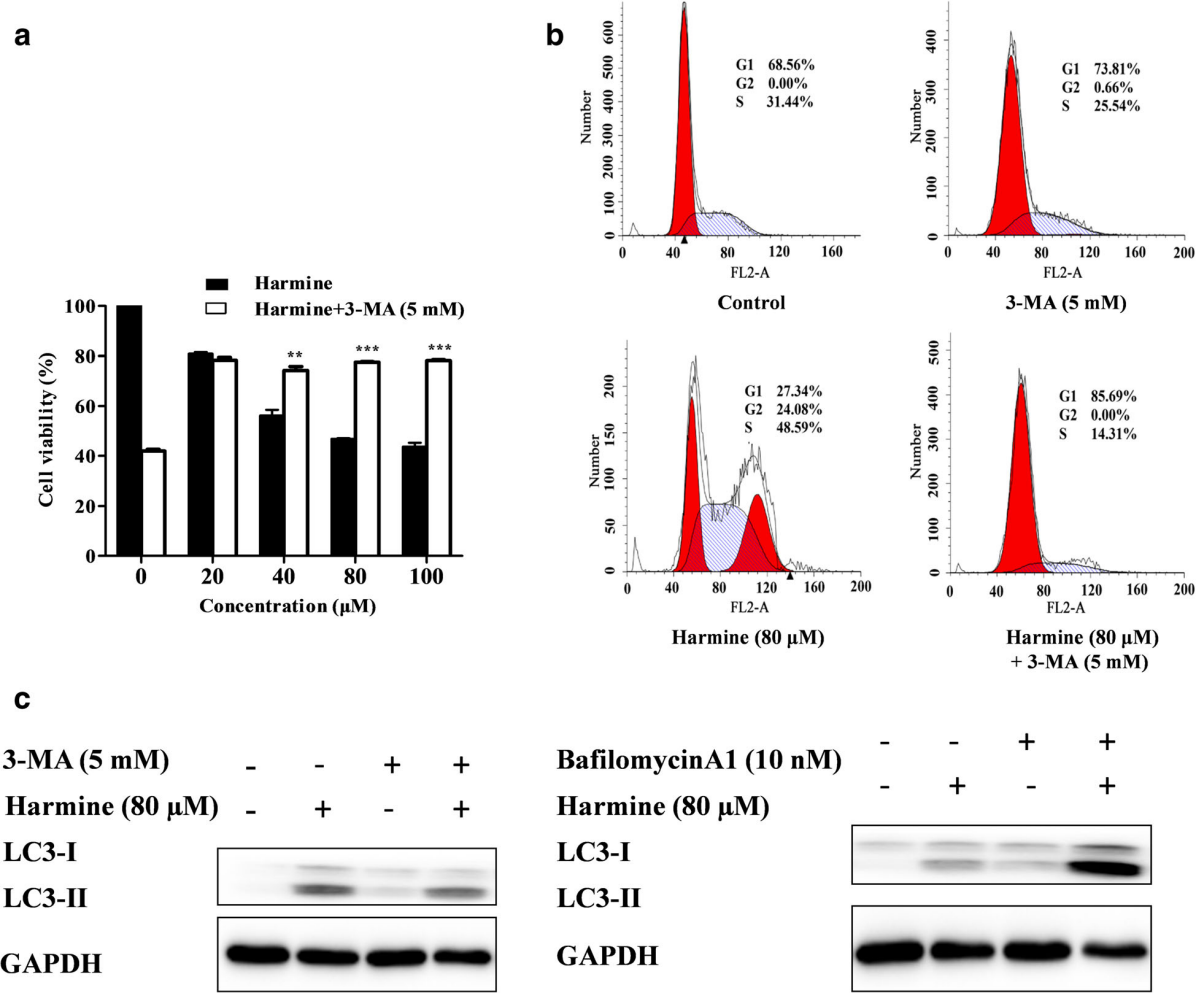
Harmine affected autophgic flux and the relation of autophagy and apoptosis. **a:** The effects of 3-MA (5 mM) on harmine induced B16 cells viability; **b:** The cell cycle distribution of B16 cells treated with harmine (80 μM) and combine with 3-MA (5 mM) for 24 h; and **c:** the protein expression of LC3-II of B16 cells treated with harmine (80 μM) and combine with 3-MA (5 mM) or bafilomycin A1 (10 nM)

The cell viability of co-administrated harmine and 3-MA was shown in Fig. 5a. Following addition of PI3-kinase inhibitor 3-MA (5 mM), the cell viability was increased compared with corresponding harmine-treated cells.

For the cell cycle of co-administrated harmine and 3-MA, it was observed that 3-MA (5 mM) resulted in an expected reduction in autophagosome formation, which caused a clear decrease in cell cycle arrest of G2 phase compared with the control group as depicted in Fig. 5b. These findings prompted us to explore if the harmine-induced autophagy might has a cytotoxic effect.

In order to identifying the action site of harmine induced autophagy, the protein expression of LC3-II was examined after pretreatment with 3-MA and bafilomycin A1 for 1 h prior to the incubation with B16 cells for 24 h. 3-MA is a PI3-kinase inhibitor that can intervene autophagosome formation [27]. Bafilomycin A1 is a vacuolar H^+^-ATPase inhibitor that can avoid endosomal acidification and obstruct autophagosome-lysosome fusion [28]. As shown in Fig. 5c, the expression of LC3-II protein was slightly suppressed by 3-MA in harmine-induced cells, and bafilomycin A1 effectively increased the expression of LC3-II protein in harmine-induced cells.

### The effects on the Akt/mTOR signaling pathway

**Fig. 6:**
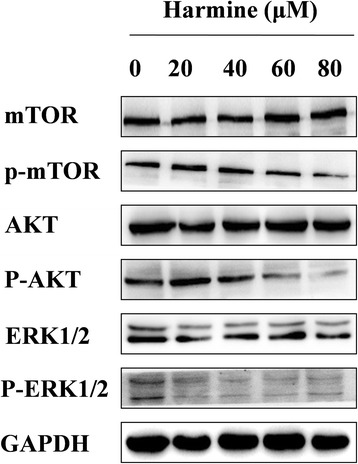
The western blot analysis for mTOR, p-mTOR, Akt, p-Akt, ERK1/2 and p-ERK1/2 signaling pathway of B16 cells induced by harmine treated with 20, 40, 60, 80 μM for 1 h

To get some insight into the molecular mechanisms of harmine-mediated autophagy induction in B16 cells, the Akt/mTOR and ERK1/2 signaling pathways were assessed. As shown in Fig. 6, treatment with harmine decreased the expression of phosphorylation of Akt and mTOR in a dose-dependent mode without influencing the total protein amount. Meanwhile, the phosphorylation levels of ERK1/2 were also inhibited without affecting the amount of total protein.

## Discussion

Although the incidence of melanoma has slowly diminished, it nowadays still is one of widely recognized cancer types in the world [29–31]. It is a highly fatal disease which consists of limited therapeutic options and poor prognosis.

This study showed that MNZQ could display potential inhibitory effects on B16 cells but not affect tyrosinase activity. The target sites of some TCM did not influence the tyrosinase activity, but might play a role on inhibiting cell proliferation of melanoma by other links of melanin metabolism. The clinical effects of MNZQ were come from the results of the compatibility of the whole 13 medicinal herbs. Alkaloids harmine, harmaline, harmane, and harmol derived from *P. harmala* were the effective ingredients in MNZQ. And harmine was confirmed as the most potent anti-melanoma agent screened from MNZQ. It could provide some evidences for the clinical effect of MNZQ on melanoma. In the previous study, it was found that harmine could significantly inhibit tumor cell proliferation and anticancer mechanism which was related to apoptotic signaling pathways [32, 33].

In harmine-treated cells, it was focused on the formation of autophagic vacuoles, whereas the nucleus remained intact. However, apoptosis is characterized by cell membrane blebbing, cell shrinkage, nuclear fragmentation, chromatin condensation, DNA fragmentation, and formation of apoptotic bodies. These findings indicated that harmine therapy induced autophagy instead of apoptosis in B16 cells. MDC has been regarded as a stalker for autophagic vacuoles. MDC-positive structures contained lysosomal enzymes, but not early/late endosomal marker. Nevertheless, the MDC dots co-localize well with staining for late-endosomal and lysosomal markers. The results of MDC staining showed that the level of autophagy increased in the dose-dependent mode. These two methods from the perspective of different morphology showed harmine induced autophagy.

From the aspects of molecular marker, the results showed that harmine induced the accretion of LC3-II and down-regulated P62 in a dose-dependent mode. The cytoplasmic form LC3-I is treated and recruited to the autophagosomes, in where LC3-II is produced by site-specific proteolysis and lipidation adjacent to the C-terminus. Therefore, there is positive correlation between the number of autophagosomes and the amount of LC3-II. A link between LC3 and ubiquitinated substrates is SQSTM1/P62 [34]. The initiation of autophagy relates with diminished doses of p62. Also, an autophagic flux assay is conducted to differentiate whether autophagosome formation is caused by autophagy induction or obstruction of the down-stream steps. Bafilomycin A1 was reported to inhibit autophagosome-lysosome fusion, expressively elevated the number of LC3-II. The accumulation of LC3-II was markedly changed in the occurrence of bafilomycin A1, suggesting that harmine increased the autophagic flux.

In addition, the rate of early and late apoptotic cells in B16 cells applied with harmine was fundamentally higher than that of control cells. In order to observe the morphological structure of apoptosis, the DNA fragmentation or condensation of the cells was examined by hoechst 33,258 staining [35]. The results revealed that the volume of nuclear enlarged and had no typical characteristic compared with the control group. It could be clearly distinguished from the typical morphologic change of apoptosis. Harmine might change the cell cycle distribution and causes G2 phase arrest. The low apoptosis rate after harmine treatment indicated that apoptosis is not a major mechanism of harmine induced cell death.

The role of autophagy in tumor is complicated and involves several opposite functions, such as promoting cell death or cell survival. As a PI3-kinase specific inhibitor, 3-MA could inhibit cell cycle arrest by blocking autophagosome formation. The data showed that combined treatment with 3-MA increased harmine-induced cell viability. Due to the change of cell cycle distribution in the G2 phase, harmine-induced cell death was not a typical apoptosis and it may be closely related to autophagy. The data provided evidences to explain the tight connection of autophagy, cell cycle distribution, and apoptosis.

Multiple signaling pathways such as AKT/mTOR, and ERK1/2 govern autophagy [36]. Akt/mTOR and ERK1/2 pathways control autophagy-induced nutrient starvation. The Akt/mTOR pathway negatively regulates autophagy [36–38] and the ERK1/2 pathway positively regulates autophagy. These pathways are also usually linked with oncogensis [39]. Some investigations have revealed that the inhibition of phosphorylation of Akt and its downstream mTOR signaling target could induce the initiation of autophagy. In this study, it was found harmine could influence Akt/mTOR and ERK1/2 signals by reducing the expression of p-mTOR, p-Akt, p-ERK1/2 in a dose-dependent manner. The results proved that harmine-induced autophagy was essentially via the Akt/mTOR pathway and in some degree through ERK1/2 pathway. Recently, it has been reported that harmine induced autophagy in MGC-803 and SGC-7901 cells by the inhibition of Akt/mTOR/p70S6K, the activation of AMPK pathway and mitochondrial pathway in human gastric cancer cells [40].

## Conclusion

The investigation demonstrated that MNZQ can inhibit cell proliferation of melanoma without inhibiting tyrosinase activity and play a key role in causing autophagy in B16 cells. Harmine could efficiently accelerate cell death and that the cell death is related to autophagy to a great extent. Moreover, harmine could activate multiple autophagy-related signaling pathways, including Akt/mTOR and ERK1/2 pathways. Harmine might be a potential cancer therapy compound for melanoma.

## Abbreviations

3-MA: 3-methyladenine
ANOVA: One-way analysis of variance
CCK-8: Cell Counting Kit-8
DMSO: Dimethyl sulfoxide
FITC: Fluorescein isothiocyanate
LC3: Microtubule-associated protein1 light chain 3
MDC: Monodansylcadaverine
MNZQ: Compound Muniziqi granule
mTOR: Mammalian target of rapamycin
PI: Propidium iodide
PI3K: Phosphatidyl inositide 3-kinase
